# From ideas to long-term studies: 3D printing clinical trials review

**DOI:** 10.1007/s11548-018-1793-8

**Published:** 2018-05-22

**Authors:** Jan Witowski, Mateusz Sitkowski, Tomasz Zuzak, Jasamine Coles-Black, Jason Chuen, Piotr Major, Michał Pdziwiatr

**Affiliations:** 1 2nd Department of General Surgery, Faculty of Medicine, Jagiellonian University Medical College, Kopernika 21 St., 31-501 Kraków, Poland; 2 Centre for Research, Training and Innovation and Surgery (CERTAIN Surgery), Kraków, Poland; 3 Human Anatomy Department, Medical University of Lublin, Jaczewskiego 4, 20-090 Lublin, Poland; 4 Department of Vascular Surgery, Austin Health, Melbourne, VIC Australia

**Keywords:** 3D printing, Clinical trials, Anatomical models, Preoperative planning, Review

## Abstract

**Purpose:**

Although high costs are often cited as the main limitation of 3D printing (3DP) in the medical field, current lack of clinical evidence is asserting itself as an impost as the field begins to mature. The aim is to review clinical trials in the field of 3DP, an area of research which has grown dramatically in recent years.

**Methods:**

We surveyed clinical trials registered in 15 primary registries worldwide, including ClinicalTrials.gov. All trials which utilized 3DP in a clinical setting were included in this review. Our search was performed on December 15, 2017. Data regarding the purpose of the study, inclusion criteria, number of patients enrolled, primary outcomes, centers, start and estimated completion dates were extracted.

**Results:**

A total of 92 clinical trials with ${N}=6$252 patients matched the criteria and were included in the study. A total of 42 (45.65%) studies cited China as their location. Only 10 trials were multicenter and 2 were registered as international. The discipline that most commonly utilized 3DP was Orthopedic Surgery, with 25 (27.17%) registered trials. At the time of data extraction, 17 (18.48%) clinical trials were complete.

**Conclusions:**

After several years of case reports, feasibility studies and technical reports in the field, larger-scale studies are beginning to emerge. There are almost no international register entries. Although there are new emerging areas of study in disciplines that may benefit from 3DP, it is likely to remain limited to very specific applications.

**Electronic supplementary material:**

The online version of this article (10.1007/s11548-018-1793-8) contains supplementary material, which is available to authorized users.

## Introduction

Despite the presence of 3D printing (3DP) in medicine for several years, many clinical centers have yet to adopt the technology as part of routine clinical care, not merely due to high costs and difficulties with segmentation—which are commonly known as the bottleneck of the process [1, 2]—but also due to lack of scientific evidence [3]. There remain insufficient studies reporting on the ways in which 3DP would affect clinical decision-making and intervention outcomes. Only a few randomized or even cohort and case-control studies are available, most of which are published in the fields of Maxillofacial Surgery and Orthopedics [4]. Cardiology emerges as another field with several valuable studies, both in interventional [5] and educational [6] aspects. Currently, clinical trials are being conducted to find new applications of this technology in other medical fields.

**Fig. 1:**
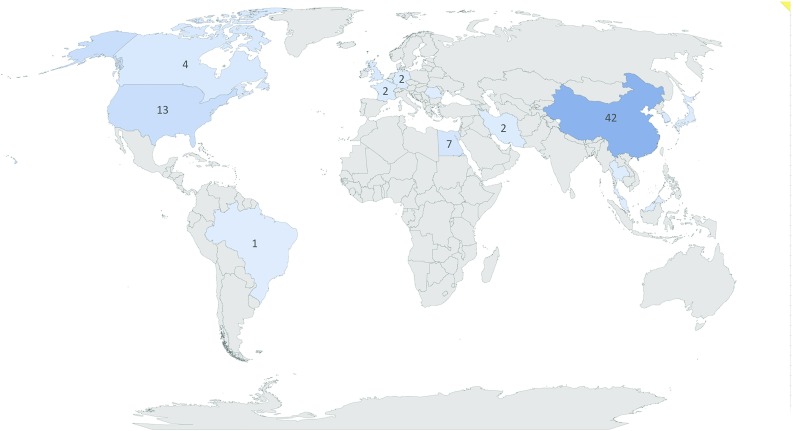
Main locations of 3DP clinical trials by country

There are several literature reviews published in the medical 3DP space. They vary from general “reviews of medical/surgical applications” [3, 4, 7] to case reports or even conference abstracts, from specific fields such as: Neurosurgery [8], Cardiology [9], Anesthesia [10] or Spinal Surgery [11]. For example, our research group previously published a systematic review on 3DP in liver surgery, evaluating all 14 papers published in this specific area [12]. Although certain fields such as Cardiothoracic Surgery already boast several reviews, there has yet to be a broader evaluation of clinical trials in 3DP, which have increased dramatically in recent years.

The purpose of this article is to review all clinical trials registered in international registries, including US National Library of Medicine ClinicalTrials.gov database and the 15 databases merged in the World Health Organization’s (WHO) International Clinical Trials Registry. To our knowledge, this is the first review of clinical trials currently interrogating the utility of 3DP.

## Materials and methods

Clinical trials registered in the ClinicalTrials.gov database, EU Clinical Trials Register and—additionally—in World Health Organization’s International Clinical Trials Registry Platform were screened. The latter includes data from 15 primary registries worldwide, including Australia and New Zealand’s ANZCTR, Chinese ChiCTR Registry, Japan’s JPRN and more. Trials that utilized 3DP by any means were included, without time, geographical or implementation limitations.

The search was performed on December 15, 2017, using the terms: “3D print*”, “additive manufacturing”, “rapid prototyping” and “bioprinting” (see Supplement 1 for full search strategy). After removing duplicates and including only studies matching the inclusion criteria, we extracted data on: purpose of the study, admission criteria, number of patients enrolled, primary outcomes, centers (including the leading and cooperating institutions and sponsors), start and estimated completion date. Papers were segregated into different fields based on their title, studied condition and description.

## Results

Our database search yielded 132 results, from which 92 matched the inclusion criteria (*N* = 6252 patients).

### Worldwide distribution

3DP clinical trials were registered in 20 different countries as main study locations (Fig. 1), most commonly in China with 42 (45.65%) trials. The next most engaged locations were USA and Egypt with 13 and 7 studies, respectively. All Egypt’s studies were located at Cairo University.

Ten trials (10.87%) are registered as multicenter, but only two (2.17%) have international collaboration: NCT02873403, a 3DP knee-brace study based in Scotland with collaborators in Germany, and NCT02846974 with main centre in Canada and an external location within Al Shifa Hospital in Gaza.

### Fields

By far, most common medical discipline that facilitates 3DP is Orthopedics with 25 (27.17%) registered trials. Other major fields are Dentistry and Maxillofacial Surgery with 13 (14.13%) and 10 (10.87%) trials, respectively (Table 1).

**Table 1:** Registered clinical trials classified by field. All results are *n* (%)

Field	# of trials (${n}=92$)
Orthopedics	25 (27.17)
Dentistry	13 (14.13)
Maxillofacial surgery	10 (10.87)
ENT	7 (7.61)
Oncology	7 (7.61)
Ophthalmology	5 (5.43)
Cardiology/Cardiac surgery	4 (4.35)
Urology	4 (4.35)
Neurosurgery	4 (4.35)
General surgery	3 (3.26)
Others/Non-classified	10 (10.87)

### Patient recruitment

At the time of data extraction, 43 (46.74%) trials were in the process of enrolling patients in their studies. 17 (18.48%) were already completed, 15 (16.3%) had yet to commence recruiting patients, and 12 (13.04%) were still pending. There were 4 (4.35%) studies with the “unknown” status and one (1.09%) marked as active, but not recruiting.

### Timeline and recruitment status

**Fig. 2:**
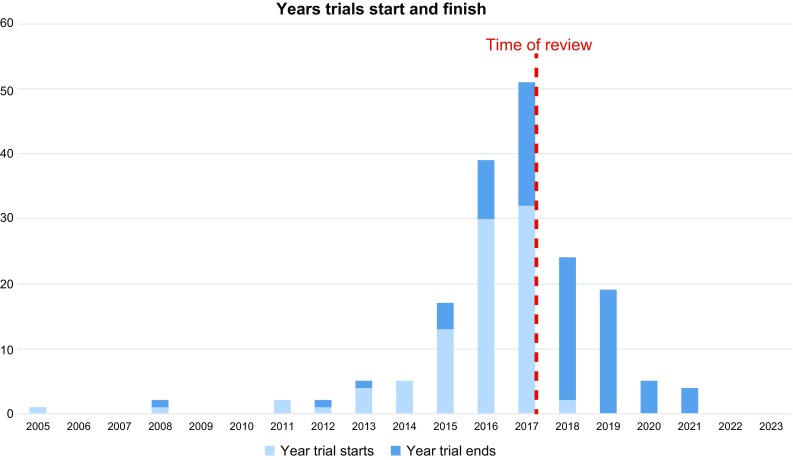
Chart displaying number of trials starting and ending each year from 2005 to 2023

In our analysis, 89 (96.74%) studies were projected to commence before 2018—32 (34.78%), of which in 2017, 30 (32.61%) in 2016 and 13 (14.13%) in 2015. 2 trials are planned to commence in 2018. Most trials were projected to finish in 2018, 2017 and 2019, with 22 (23.91%), 19 and 19 (20.65%) studies, respectively (Fig. 2).

### Implementations

Due to large diversity of 3DP implementations in included trials, it is not possible to perform any sort of statistical or pooled analysis. We have described several examples of 3DP applications in Table 2.

**Table 2:** Examples of implementations of 3DP in included clinical trials for various medical fields

Field	Clinical trial registration #	Implementation
Orthopedics	NCT03185286	Personalized metal implants for bone defect surgeries
	NCT02873403	3DP knee brace compared to conventional knee brace
	NCT02845245	3DP prototype for preoperative planning in patients with distal tibia fractures
Maxillofacial surgery	NCT02914431	Personalized 3DP titanium plates vs. traditional surgical splints in maxillary repositioning of orthognathic surgery
	ChiCTR-ONC-17010475	3D CT-based bone model for reconstruction of orbital-maxillary-zygomatic complex
	ChiCTR-INR-16009695	Personalized, 3DP nasal bone positor for nasal bone fracture treatment
Dentistry	NCT03281603, NCT03119753, NCT03354715, NCT01191073	3DP vs. conventional complete dentures
ENT	NCT03111888	Patient-specific silicone stent airway implant for chronic obstructive pulmonary disease patients
	NCT02905344	3DP anatomical models to educate patients before deviated nasal septum surgery
	NCT02559050	Case of 3DP nasal stent for congenital arrhinia
Oncology	NCT02952261	CT-based, 3DP device to localize small pulmonary nodule and indicate puncture site
	NCT02550210	Breast Cancer Locator—3DP bra-like form for intraoperative guidance
Ophthalmology	IRCT2017042633094N1	Personalized template for orbital floor fracture reconstruction guidance
	NCT01312545	3DP versus conventional orbital implant for enucleation
Cardiology and Cardiac surgery	NCT03330210	Patient-specific left atrial appendage models for pre-procedural device sizing and planning
	ChiCTR-IPR-17012001	3D model and computer-based simulations versus traditional imaging evaluation in planning treatment of right-sided congenital heart disease
Urology	ChiCTR-ONC-16010296	3DP extravascular stent placed laparoscopically in patients with nutcracker syndrome
	NCT03272529	Models for rehearsal of percutaneous nephrolithotomy
Neurosurgery	ChiCTR-IOC-16009978	Simulation of intracranial aneurysm treatment
General surgery	ISRCTN75603704	Patient-specific models for rectal cancer surgery rehearsal

## Discussion

This review, which surveys 92 registered clinical trials with a projected enrollment of over 6000 patients, displays the rapid growth of the field, its challenges, its current direction, and how 3DP will be utilized by clinicians in the coming years.

There is a surge in number of trials registered after 2015, especially in 2016 and 2017. This clearly displays the transition of 3DP from its early phases of end-users evaluating its feasibility, to the next phase of serious implementation, routine use and clinical trials.

The IDEAL framework divides innovations in surgery and interventional procedures into five phases: (1) idea, (2) development, (3) exploration, (4) assessment and (5) long-term study [13, 14]. As elucidated by our review, this process clearly describes the current shifts in the field of clinical 3DP. Prior to 2015, most clinicians were still postulating on the future applications of 3DP, next steps and how it should be applied. This corresponds with the “idea” and “development” stages of the IDEAL framework [15–17]. The significant number of publications and—as displayed in our review—clinical trials, confirms the advance toward the “long-term study” phase for the field.

This is especially important when considering the current limitations stopping this technology from broader use. Typically, the barriers cited are those related to segmentation, high costs and printing time [18]. The current lack of empirical evidence is another significant barrier toward the broader adoption of the technology by more conservative clinicians. As elucidated in our review, we project that several dozen registered studies will publish their results in 2018–2020, which will objectively demonstrate or rebut the feasibility and clinical utility of 3DP. To date, these trials are largely limited to only few fields: primarily Orthopedic Surgery, Maxillofacial Surgery and Dentistry. Given the current state of the field, this is unsurprising, but change may very well be afoot. A systematic review by Martelli et al. from 2016 showed that 75% of all medical applications of 3DP are in the fields of Orthopedics and Maxillofacial Surgery [3]. A similar publication by Tack et al.—inclusive of Dental publications— displayed that greater than 50% of papers reside in these two fields [4]. However, at the time of Tack’s study, only 4.82% of papers were in the field of Dentistry, a percentage which has increased significantly by the time of our review.

Dental clinical trials most often evaluate 3D-printed complete (NCT03281603, NCT03354715, NCT03119753) and partial (NCT01191073, NCT01191073, ChiCTR-ONC-16009899, IRCT2015022221190N1) dentures. In Orthopedics and Maxillofacial Surgery, the use of 3DP implants is of import. For instance, a large (300 patients) NCT03166917 trial will evaluate the clinical outcomes after using personalized 3DP implants to treat bone defects. With 3 centers participating, this trial is one of the largest running and is projected to finish in 2021. Other implementations in Orthopedic Surgery include orthoses (ChiCTR-OIC-17013130) and surgical templates (ChiCTR-INR-16009961)

There are some very promising studies outside those fields, i.e., in Cardiology and Cardiac Surgery. In fact, French study NCT03330210 (LAA-PrintRegis)—designed to start in December 2017—will evaluate the outcomes of utilizing 3DP in left atrial appendage closure, enrolling 400 patients in 13 French institutions. ChiCTR-IPR-17012001, also including 400 subjects, aims to assess 3DP as adjunctive tool for the diagnosis and treatment of right-sided congenital heart malformations. Its projected completion date is 2020. These trials would be a great addition to current state-of-the-art literature in this field, which has already shown 3DP to be beneficial for planning various interventions [19, 20]. Even fully personalized tools, including patient-specific occluders, as described recently by Robinson et al., can be developed [21] with additive manufacturing.

The clear role that 3DP can play in anatomical visualization lends itself toward certain applications more so than others, emphasized by the fact that multiple trials are currently registered to treat specific conditions (i.e., edentulous jaw, bone fractures, left atrial appendage occlusion, aneurysms, congenital heart defects). On the other hand, other disciplines may not be as suited to this technology, where alternatives such as virtual or augmented reality may be a better fit [22].

In conducting this review, we are aware of its limitations. For instance, there exists clinical trials that are not registered in the databases we have surveyed in our methodology. Nevertheless, the 92 studies we have compiled are sufficient to provide commentary on the current state of the field. In addition, some registered trials have “unknown” as their database status and may not have been updated for some time. Finally, this review cannot comment on how 3DP affects clinical decision-making, as we have yet to evaluate the results of completed studies. A meta-analysis in the coming years would be prudent to evaluate specific techniques, such as left atrial appendage closure, where sufficient interventions will have been completed to power statistical analysis. This requires authors to publish substantiated, quantitative results, which has been an issue in the past. Very often clinicians are focused only on their opinions and qualitative results. Additionally, it might be obvious for some people that improved, advanced three-dimensional visualizations offered by 3DP are beneficial over traditional approach. Still, we will not be able to implement the technology widely without analysis of cost-effectiveness and facts about its impact on clinical outcomes.

As active researchers in this field, it is exciting to see the upswing in 3DP clinical trials being registered, as it is an encouraging sign that the field is moving beyond its infancy. After an initial period of gradual uptake of the technology and gradual accumulation of evidence, we may soon witness routine clinical implementation in a greater number of institutions, as its cost-effectiveness improves. The next pertinent steps for medical 3DP going forward will be to build up a larger body of empirical evidence.

## Conclusions

This study displays the growing number of 3DP clinical trials registered to international databases. Most are in the fields of Orthopedics, Dentistry and Maxillofacial Surgery, but there are emerging fields including ENT Surgery, Oncology and Ophthalmology. The current paucity of international multi-institutional trials highlights the current immaturity of the field. Sixty registered trials are projected to be complete prior to 2020, which would aid in the implementation of 3DP in routine medical practice, should the outcomes support its use. Despite this, implementation will likely only become commonplace in medical disciplines where the benefits of this technology will complement routine clinical practice.

## Electronic supplementary material

Below is the link to the electronic supplementary material.

Supplementary material 1 (docx 14 KB)
