# Supplementary material for: From ideas to long-term studies: 3D printing clinical trials review
Source: Int J Comput Assist Radiol Surg. 2018 May 22;13(9):1473–8. doi: 10.1007/s11548-018-1793-8 (PMC6132399; doi:10.1007/s11548-018-1793-8)
Supplement: Supplementary file 1 — Supplementary material 1 (docx 14 KB) [file 11548_2018_1793_MOESM1_ESM.docx]

**Supplementary Table 1. Search strategy and results from different search engines (direct NCT, ANZCTR, EU Registry and WHO International Database).**

| Database | Search Query | Results |
| --- | --- | --- |
| NCT DIRECT SEARCH | 3d print* | 38 |
| NCT DIRECT SEARCH | Additive manufacturing | 4 |
| NCT DIRECT SEARCH | Rapid prototyping | 12 |
| NCT DIRECT SEARCH | Bioprinting | 0 |
| ANZCTR DIRECT SEARCH | All | 0 |
| EU Registry DIRECT SEARCH | All | 0 |
| WHO | 3D print* | 65 |
| NCT | 3D print* | 26 |
| KCT | 3D print* | 4 |
| ChiCTR | 3D print* | 28 |
| IRCT | 3D print* | 1 |
| ISRCTN | 3D print* | 3 |
| JPRN | 3D print* | 2 |
| DRKS | 3D print* | 1 |
| WHO | Additive manufacturing | 2 |
| ChiCTR | Additive manufacturing | 1 |
| ISRCTN | Additive manufacturing | 1 |
| WHO | Rapid prototyping | 10 |
| NCT | Rapid prototyping | 6 |
| PACTR | Rapid prototyping | 1 |
| ChiCTR | Rapid prototyping | 1 |
| IRCT | Rapid prototyping | 1 |
| DRKS | Rapid prototyping | 1 |
| WHO | Bioprinting | 1 |
| ChiCTR | Bioprinting | 1 |
|  | **TOTAL** | **132** |
